# Aromatic Bromination Abolishes the Psychomotor Features and Pro-social Responses of MDMA (“Ecstasy”) in Rats and Preserves Affinity for the Serotonin Transporter (SERT)

**DOI:** 10.3389/fphar.2019.00157

**Published:** 2019-02-28

**Authors:** Patricio Sáez-Briones, Vicente Castro-Castillo, Gabriela Díaz-Véliz, Luis Valladares, Rafael Barra, Alejandro Hernández, Bruce K. Cassels

**Affiliations:** ^1^Laboratory of Neuropharmacology and Behavior, Faculty of Medical Sciences, School of Medicine, Universidad de Santiago de Chile, Santiago, Chile; ^2^Department of Organic Chemistry and Physical Chemistry, Faculty of Chemical and Pharmaceutical Sciences, Universidad de Chile, Santiago, Chile; ^3^Faculty of Medicine, Institute for Biomedical Sciences, University of Chile, Santiago, Chile; ^4^Laboratory of Hormones and Receptors, Instituto de Nutrición y Tecnología de los Alimentos (INTA), Universidad de Chile, Santiago, Chile; ^5^Laboratory of Neurobiology, Department of Biology, Faculty of Chemistry and Biology, Universidad de Santiago de Chile, Santiago, Chile; ^6^Chemobiodynamics Laboratory, Department of Chemistry, Faculty of Sciences, Universidad de Chile, Santiago, Chile

**Keywords:** MDMA (3,4-methylenedioxymethamphetamine), 2-Br-4, 5-MDMA (2-bromo-4, 5-methylenedioxymethamphetamine), rat behavior, human platelet aggregation, ATP release, serotonin transporter

## Abstract

The entactogen MDMA (3,4-methylenedioxy-methamphetamine, “Ecstasy”) exerts its psychotropic effects acting primarily as a substrate of the serotonin transporter (SERT) to induce a non-exocytotic release of serotonin. Nevertheless, the roles of specific positions of the aromatic ring of MDMA associated with the modulation of typical entactogenic effects, using analogs derived from the MDMA template, are still not fully understood. Among many possibilities, aromatic halogenation of the phenylalkylamine moiety may favor distribution to the brain due to increased lipophilicity, and sometimes renders psychotropic substances of high affinity for their molecular targets and high potency in humans. In the present work, a new MDMA analog brominated at C(2) of the aromatic ring (2-Br-4,5-MDMA) has been synthesized and pharmacologically characterized *in vitro* and *in vivo*. First, binding competition experiments against the SERT-blocker citalopram were carried out in human platelets and compared with MDMA. Besides, its effects on platelet aggregation were performed in platelet enriched human plasma using collagen as aggregation inductor. Second, as platelets are considered an appropriate peripheral model for estimating central serotonin availability, the functional effects of 2-Br-4,5-MDMA and MDMA on ATP release during human platelet aggregation were evaluated. The results obtained showed that 2-Br-4,5-MDMA exhibits higher affinity for SERT than MDMA and fully abolishes both platelet aggregation and ATP release, resembling the pharmacological profile of citalopram. Subsequent *in vivo* evaluation in rats at three dose levels showed that 2-Br-4,5-MDMA lacks all key MDMA-like behavioral responses in rats, including hyperlocomotion, enhanced active avoidance conditioning responses and increased social interaction. Taken together, the results obtained are consistent with the notion that 2-Br-4,5-MDMA should not be expected to be an MDMA-like substrate of SERT, indicating that aromatic bromination at C(2) modulates the pharmacodynamic properties of the substrate MDMA, yielding a citalopram-like compound.

## Introduction

**FIGURE 1:**
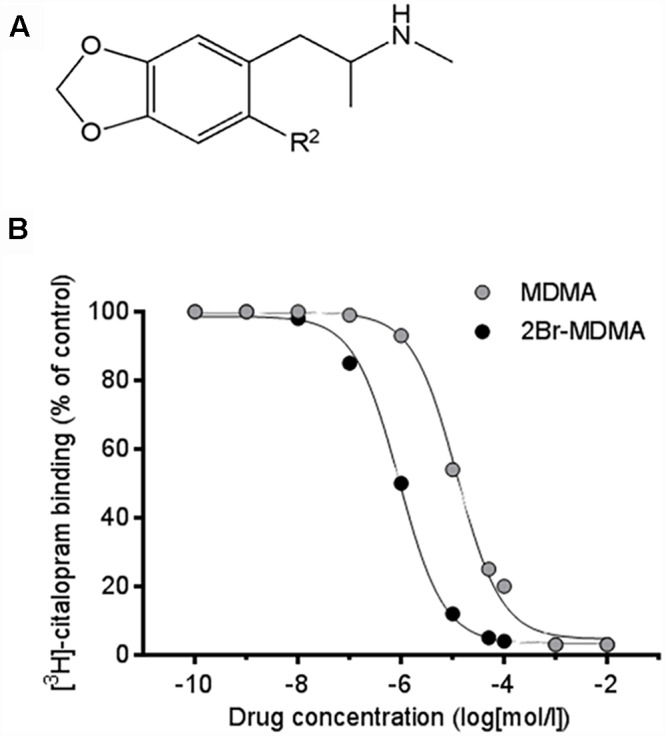
**(A)** Chemical structures illustrating the MDMA moiety. For MDMA, the substituent located at the R^2^ aromatic position is a hydrogen atom (*R*^2^ = H). For 2-Br-4,5-MDMA, the substituent located at the R^2^ aromatic position is a bromine atom (*R*^2^ = Br). **(B)** Inhibition of specific [^3^H]-citalopram binding to human platelet membranes by MDMA and 2-Br-4,5-MDMA, allowing the calculation of inhibition constant (K_i_) values (means ± SEM) from three separate experiments. For MDMA, K_i_ = 9880 ± 1231 nM; for 2-Br-4,5-MDMA, K_i_ = 1060 ± 50.2 nM (*p* < 0.002, two-tailed unpaired Student’s *t*-test). Reference values of K_i_ for serotonin and citalopram were 870 and 0.42 nM, respectively (curves not shown).

The entactogen MDMA (3,4-methylenedioxymethamphetamine, Figure 1A), also known as “Ecstasy,” engenders in humans an altered state of consciousness described as a feeling of heightened self-acceptance and empathy with other persons without impairing cognitive or orientation capabilities, while decreasing fear responses (Green et al., 2003). Due to these properties, evidence has accumulated regarding the potential applications of MDMA in psychotherapy (Parrott, 2007; Mithoefer et al., 2011, 2013; Feduccia and Mithoefer, 2018), as an adjunct in the treatment of neuropsychiatric disorders (Riedlinger and Riedlinger, 1994; Doblin, 2002; Bouso et al., 2008; Danforth et al., 2016) and alcoholism (Sessa, 2018).

MDMA is known to exert its acute psychotropic effects acting mainly as a special type of substrate of the serotonin transporter (SERT) (Crespi et al., 1997; Rothman et al., 2001) that induces non-exocytotic serotonin (5-HT) release by triggering a reversal of the normal transporter flux (Sitte and Freissmuth, 2010). As the basis of MDMA-like activity deserves to be explored for therapeutic purposes, efforts to develop MDMA analogs included mainly classical derivatives (Shulgin and Shulgin, 1991) and more recently synthetic cathinones (Morris, 2010) and benzofurans (Liechti, 2015). The group of “classical” analogs (some of them viewed as true MDMA substitutes), included the recreationally popular *N-*demethylated MDMA analog (and primary metabolite) MDA (Gouzoulis-Mayfrank and Hermle, 1998; Baylen and Rosenberg, 2006). As for the newer synthetic cathinones and benzofurans, modifications were focused on the aminoalkyl side chain, replacing the α- and the *N*-methyl by an α- (MBDB) or *N*-ethyl group (MDEA) or by locking its conformation in a 2-aminoindan ring structure (Nichols et al., 1986). Interestingly, it was further found that the ring-methylated MDA derivatives 1-(2-methyl-3,4-methylenedioxyphenyl)- and 1-(3-methyl-4,5-methylenedioxyphenyl)-2-aminopropane are not only fairly potent 5-HT releasers in rats but also substitute, at low doses, for the entactogen-like MBDB and MMAI in the drug discrimination paradigm, whereas the positional isomer 1-(2-methyl-4,5-methylenedioxyphenyl)-2-aminopropane is four times less potent than MMAI and only substitutes partially for MBDB (Parker et al., 1998). This evidence suggests that rational modifications of the benzene ring of MDMA can lead to potentially new analogs sharing some of the distinctive MDMA-like effects. As bromine can be considered as bio-isosteric to the methyl group, one should expect that appropriately brominated analogs of MDMA could exhibit similar MDMA-like properties. In addition, because halogenation has been described to modulate the interactions of a drug with its molecular target by establishing so-called “halogen bonds” (Hardegger et al., 2010), bromination might be a useful tool to reveal some valuable hints about the modulation of MDMA activity as a consequence of its interaction with SERT. Nevertheless, and despite its relevance, the pharmacological characterization of the effects of aromatic halogenation on the mode of binding at SERT referred to MDMA remains fragmentary and incomplete. For instance, aromatic bromination at C(2) to afford 1-(2-bromo-4,5-methylendioxyphenyl)-2-methylaminopropane (2-Br-4,5-MDMA) has been proposed as an approach to an analog that might exhibit entactogenic-like properties (Sáez-Briones and Hernández, 2013). This notion is supported by some members of the heterogenous group of psychotropic phenylalkylamines, including MDMA. Indeed, aromatic halogenation of structurally-related phenylalkylamine molecules can render hallucinogens of high potency *in vivo* (Barfknecht and Nichols, 1971; Shulgin and Shulgin, 1991) and high affinity for their corresponding molecular targets (e.g., 5-HT_2A/2C_ receptors; Nichols, 2016, 2018), though only when the halogen is located at C(4). The *N*-unmethylated analog of 2-Br-4,5-MDMA, 1-(2-bromo-4,5-methylendioxyphenyl)-2-aminopropane (2-Br-4,5-MDA) was prepared long ago and tested in humans, and the subjective effect of an oral dose in excess of 300 mg was considered “amphetamine-like” (Sepúlveda et al., 1972). Regardless of the fact that the latter evidence should not necessarily extend to monoamine transporters, one could expect that aromatic bromination might induce similar effects on the mode of binding of MDMA. Consequently, we hypothesize that bromine substitution may increase the pharmacological features of MDMA, including SERT affinity, the ability to act as a SERT substrate and the distinctive behavioral effects already known for Ecstasy.

To verify this hypothesis, 2-Br-4,5-MDMA has been synthesized and pharmacologically evaluated in order to determine its binding affinity at human SERT. For this purpose, binding competition experiments against the SERT blocker citalopram were carried out in human platelets and compared with MDMA. In addition, the effects on platelet aggregation were performed in platelet enriched human plasma using collagen as aggregation inductor. As platelets also possess a high SERT density, they are considered a suitable peripheral model to estimate central serotonin availability from the periphery and the amount of ATP released during platelet aggregation may be used as an indirect estimate of the activation level of SERT (Maurer-Spurej, 2005; Mercado and Kilic, 2010). Therefore, the functional features of 2-Br-4,5-MDMA on SERT were also determined evaluating the effects of the analog on platelet ATP release.

Finally, 2-Br-4,5-MDMA was evaluated *in vivo* at different doses using a sequence of behavioral paradigms in rats already used to construct the distinctive behavioral profile of MDMA (Quinteros-Muñoz et al., 2010): spontaneous behaviors (motor activity, locomotion, rearing, grooming, head shakes), evaluation in the elevated plus-maze, determination of active avoidance conditioning responses and assessment of social interaction (Sáez-Briones and Díaz-Véliz, 2012). Both drugs were tested as racemic mixtures, considering that this is the form commonly used in clinical and recreational settings. The results obtained support the notion that aromatic bromination at C(2) changes the mode of binding at SERT and the distinctive behavioral effects of MDMA.

## Materials and Methods

### General Procedures

All reagents and solvents were commercially available from Sigma-Aldrich (St. Louis, MO, United States) or Merck (Darmstadt, Germany) and were used without further purification. Melting points are uncorrected and were determined with a Reichert Galen III hot plate microscope equipped with a DUAL JTEK Dig–Sense thermocouple thermometer. ^1^H NMR spectra were recorded at 400 MHz on a Bruker AMX 400 spectrometer, using D_2_O as solvent. The chemical shifts are reported as *δ* (ppm) downfield from TMS for ^1^H NMR. Coupling constants (*J*) are given in Hz.

### Organic Synthesis of MDMA and 2-Br-4,5-MDMA

3,4-Methylenedioxybenzaldehyde was converted to the *β*-nitrostyrene (Henry-Knoevenagel condensation with nitroethane) in an ionic liquid (2-hydroxyethylammonium formate) (Bicak, 2005; Alizadeh et al., 2010). After purification by crystallization, the reaction product was reduced to the amine with LiAlH_4_. Then, 1.0 g of the 4,5-methylenedioxyamphetamine MDA (5.6 mmol) obtained was dissolved in THF (5.0 mL) and added dropwise with stirring to a solution of formic acetic anhydride (44.8 mmol) in THF (15 mL). The reaction was held at room temperature for 6 h. All volatile components were removed under vacuum affording a red colored oil, that was treated without purification with LiAlH_4_ (1.0 g) in THF (10 mL). Then, 500 mg of the MDMA (Figure 1A) obtained was dissolved in AcOH (2.0 mL) and a 4 mol/L solution of Br_2_ in AcOH (2.8 mmol) was added dropwise with stirring. The reaction was kept at room temperature for 24 h. The precipitate formed was collected by filtration to afford 2-Br-4,5-MDMA.HBr (Figure 1A), which was obtained as white crystals (1.2 g, 63%); mp 196–197°C. ^1^H NMR (400 MHz, D_2_O): δ 1.21 (d, *J* = 6.6 Hz, 3H, CH_3_), 2.66 (s, 3H, CH_3_), 2.93 (m, 2H, CH_2_), 3.48 (m, 1H, CH), 5.92 (s, 2H, CH_2_), 6.78 (s, 1H, ArH), 7.04 (s, 1H, ArH).

### Binding of MDMA and 2-Br-4,5-MDMA at 5-HT Transporters (SERT)

Binding of MDMA and 2-Br-4,5-MDMA at SERT was determined from competition curves of the drugs against the high affinity ligand [^3^H]-citalopram, using a modification of the assay in platelet membranes as described (Plenge and Mellerup, 1991). Blood from healthy donors was collected by venipucture into acid-citrate-dextrose (9:1) and centrifuged at 200 ×*g* for 20 min to prepare platelet rich plasma (PRP). PRP (5 mL) was diluted in 20 mL buffer A (50 mM Tris–HCl buffer pH 7.4 containing 120 mM NaCl and 5 mM KCl) and centrifuged at 1700 ×*g* for 20 min. The supernatant was discarded and the final membrane pellet was homogenized in 10 mL of buffer A and centrifuged twice at 27,000 ×*g* for 20 min. It was then resuspended in 10 mL buffer A to yield a final protein concentration of about 0.8–1.2 mg/mL. [^3^H]-citalopram binding was determined in 200 μL of platelet membranes loaded with 100 μL [^3^H]-citalopram (2 nM). This concentration is in the range of the dissociation constant (K_D_) value for citalopram binding at SERT in PRP (Plenge and Mellerup, 1991). Fifty micro liter of buffer (50 mM Tris-HCl, pH 7.4) containing increased concentrations (0.1 nmol/L to 10 mmol/L) of the unlabeled drugs (i.e., MDMA, 2-Br-4,5-MDMA), serotonin or citalopram was then added. After 60 min incubation at 25°C, homogenates were diluted in 3 mL ice-cold buffer and filtered through Whatman GF/C glass fiber filters. The total time taken for the filtration/washing procedure was less 30 s. Filters were washed three times with 3 mL ice-cold buffer, and the radioactivity was measured by liquid scintillation spectrometry at 55% efficiency. Specific binding was defined as the difference between total labeled [^3^H]-citalopram binding (triplicate samples) and the binding in the presence of 10 μM unlabeled citalopram (duplicate samples). The inhibitory constants (K_i_), defined as the drug concentration displacing 50% of the specifically bound [^3^H]-citalopram, were calculated from one-site competitive binding data using the Prism 5.0 program (GraphPad Inc., San Diego, CA, United States) according to Cheng and Prusoff (1973), and considering a K_D_ = 1.7 nM for [^3^H]-citalopram binding in human platelets (Plenge and Mellerup, 1991).

### Effects of MDMA and 2-Br-4,5-MDMA on Platelet Aggregation and SERT Functionality

The effects of MDMA and 2-Br-4,5-MDMA on SERT functionality were indirectly determined by studying the release of ATP during platelet aggregation induced by collagen, which is a process associated with 5-HT uptake at SERT. PRP were prepared from blood collected from human healthy donors as described above, and platelets were pelleted by centrifugation at 500 ×*g* for 10 min and washed and re-suspended as described (Kim et al., 2013). Both platelet aggregation and ATP release from platelets were simultaneously measured using a lumi-aggregometer (Chrono-Log, United States). PRP were incubated and stabilized at 37°C in an aggregometry sample tube. Each sample was stirred at 1000 rpm for 1 min before testing. Samples were further pretreated in the presence of different concentrations (12.5, 25, 50, or 100 μM) of 2-Br-4,5-MDMA or MDMA for 10 min. Platelet aggregation was then induced by addition of collagen (1 μg/mL) as previously described (Muñoz et al., 2012). The resulting aggregations were measured as changes of light transmission that were recorded for 8 min. The extent of platelet aggregation was expressed as percentage of light transmission in a platelet-free medium. The ATP released from platelets was detected by a luciferin-luciferase detection system, ATP Bioluminescent Assay Kit (Sigma-Aldrich), according to the manufacturer’s directions, and expressed as percentage.

### Behavioral Evaluation

#### Animals

A total of 80 adult male Sprague-Dawley rats, weighing 200–230 g, were purchased from the rearing facility of the Pontifical Catholic University of Chile, and housed eight per cage in a temperature-controlled vivarium under a 12:12 h light-dark cycle (lights on from 0800 to 2000 h), with free access to standard rodent pellet diet and tap water. Behavioral observations took place in a sound-proof room at the same time of day to reduce the confounding influence of diurnal variation on spontaneous behavior. Each animal was tested only once, and the minimum number of animals and duration of observations required to obtain consistent data were employed. Experimental protocols were conducted in accordance with international standards of animal welfare and following the Guide for Care and Use of Laboratory Animals, National Research Council, United States, and were approved by the Bioethics Committee of the University of Santiago de Chile.

#### Drug Administration

Each drug was freshly dissolved in saline (0.9% NaCl) and administered intraperitoneally (i.p.) in a volume of 1 mL/kg body weight. MDMA and 2-Br-4,5-MDMA were administered at dose levels of 1, 5, and 10 mg/kg (i.p.), 30 min before the behavioral tests. Each experimental group consisted of 6–8 animals. Saline was used as control treatment. Certificate No 120, Institutional Ethics Committee, Universidad de Santiago de Chile.

#### Spontaneous Motor Activity

Each rat was placed individually in a Plexiglas cage (30 × 30 × 30 cm) located inside a soundproof chamber. The floor of the cage was an activity platform (Lafayette Instrument Co., Lafayette, IN, United States) connected to an amplifier and an electromechanical counter to monitor total spontaneous motor activity (Lafayette Instrument Co., United States). Locomotor activity was also recorded with an infrared photocell activity monitor (Columbus Instruments, United States), provided with an array of 15 infrared photocells spaced 1 in. (2.54 cm) apart. Total motility and locomotor activity were monitored every 5 min during a 30 min period. Simultaneously, the number of rearings, head shakes and the time (in seconds) spent in grooming behavior were manually recorded. Global spontaneous motor activity measures the total motor activity of the animal, including grooming, head shakes, rearing and locomotion. In contrast, locomotor behavior measures the horizontal displacement of the animal only. One head shake was scored when the animal exhibited a rapid up-and-down and/or rotating motion of the head, sometimes affecting the trunk as in “wet-dog shakes.” One spontaneous grooming episode was scored as time spent by the animal in wiping and/or licking different body parts. All the observations were recorded in real time using a digital camera connected to a PC, and video sequences were used for later reanalysis when necessary.

#### Elevated Plus-Maze

This test has been widely validated to measure anxiety in rodents (Pellow and File, 1986). The apparatus consisted of two black Plexiglas open arms (50 × 10 cm each), two closed arms (50 × 10 × 20 cm each) and a central platform (10 × 10 cm). The maze was elevated 70 cm above the floor. Each animal was placed at the center of the maze, facing one of the closed arms. During a test period of 5 min, an observer recorded: (a) the number of open-arm entries; (b) the number of closed-arm entries; (c) the time spent in open arms; and (d) the time spent in closed arms. Arm entries were counted when the animal placed all four paws in an arm. Because illumination seems to play a crucial role in the plus-maze behavior of rats (Mora et al., 1996), the test was conducted under low artificial illumination conditions (approximately 10 lux). After the test, the maze was carefully cleaned with a wet tissue paper (70% ethanol solution). The results were expressed as percentages of open-arm entries and of time spent in open arms, with regard to the total number of arm entries and the total time spent in both open and closed arms, respectively. Since, in this test, anxiety is reflected in the unconditioned aversion to heights and open spaces, the percentage of entries and time spent in open arms provide measures of fear-induced inhibition of exploratory activity. This ratio is increased by anxiolytic and reduced by anxiogenic compounds (Pellow et al., 1985).

#### Active Avoidance Conditioning

Each rat was individually placed into a two-way shuttle box (Lafayette Instrument Co., Lafayette, IN, United States) composed of two stainless steel modular testing units. Each unit was equipped with an 18-bar insulated shock grid floor, two 28 V DC lights and a tone generator (Mallory Sonalert 2800 Hz, Lafayette Instrument Co., Lafayette, IN, United States). Electric shocks were delivered to the grid floor by a master shock supply (Lafayette Instrument Co., Lafayette, IN, United States). The rats were trained over 50 trials, after a 5 min period of habituation. Each trial consisted of the presentation of a tone that after 5 s was overlapped with a 0.20 mA foot-shock until the animal escaped to the opposite chamber (maximum shock duration of 10 s). Between trials, the animal was allowed to rest for at least 15 s. A conditioned avoidance response (CAR) was defined as a crossing to the opposite chamber within the first 5 s (tone alone). If the rat did not escape by crossing to the opposite chamber during the foot-shock, this was considered as an escape failure (EF).

#### Assessment of Social Interaction

To evaluate social interaction, a modification of the method described by File (1980) was employed. A pair of Sprague Dawley rats (220–250 g) coming from different cages (i.e., without previous interaction) were marked on the tail with two different non-permanent dye colors and injected with a single dose (2.5 mg/kg or 5 mg/kg, i.p.) of either MDMA or 2-Br-4,5-MDMA. Rat pairs injected with saline i.p. served as controls. Thirty minutes after the injection, the rat pair was placed in an open black box (50 × 50 × 40 cm) with high walls made of acrylic polymer, and the spontaneous interaction in the test arena was observed and scored during 10 min. To do this, the time (in seconds) spent on diverse behaviors related to social interaction (approach and remaining side-by-side with the partner, general and anogenital sniffing of the partner) and the number of rearings and times lying belly down were scored independently for the two rats by two experienced persons. The test was conducted under low artificial illumination conditions (approximately 10 lux), and all observations were recorded in real time using a digital camera connected to a PC. After running the test, the box was carefully cleaned with a tissue paper wet with 70% ethanol solution.

#### Statistical Analysis

Data are presented as mean ± SEM, and all statistical analyses were performed with GraphPad Prism software (GraphPad Software, Inc., San Diego, CA, United States). For binding assays, each inhibitory constant (K_i_) obtained was compared to that calculated for citalopram, a prototypical inhibitor of SERT, using two-tailed unpaired Student’s *t*-test. For the effects on platelet aggregation, ATP release from platelets and rat behavior, data were analyzed by two-way ANOVA followed by the Dunnett (intragroup comparisons against saline control) or the Bonferroni (intergroup comparisons at similar concentrations or doses) multiple comparison *post hoc* tests. A probability level of 0.05 or less was accepted as significant.

## Results

### Effects of MDMA and 2-Br-4,5-MDMA on [^3^H]-Citalopram Binding to Platelet Membranes

Drug competition curves for [^3^H]-citalopram binding (Figure 1B) allowed the inhibition constants (K_i_) of MDMA and 2-Br-4,5-MDMA to be calculated for the site that is labeled by [^3^H]-citalopram, which most likely is SERT. The K_i_ for MDMA at SERT was 9880 ± 1231 nM, while for 2-Br-4,5-MDMA it amounted to 1060 ± 50.2 nM (*p* < 0.002, two-tailed unpaired Student’s *t*-test). Thus, the K_i_ value for 2-Br-4,5-MDMA was closely similar to that calculated for serotonin (879 nM), but it is three orders of magnitude higher than that of the classical SERT inhibitor citalopram (0.42 nM). Therefore, K_i.MDMA_ > K_i.2BrMDMA_ = K_i.5-HT_ > > K_i.citalopram_.

### Effects of MDMA and 2-Br-4,5-MDMA on Platelet Aggregation and ATP Release

**FIGURE 2:**
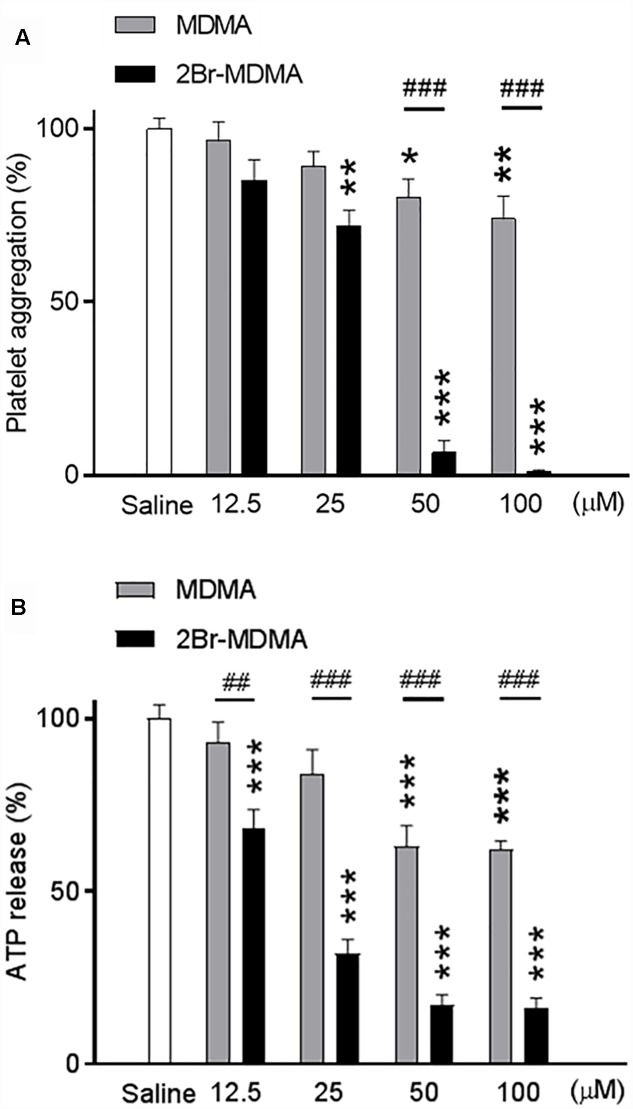
Concentration-response relationships for the effects of MDMA and 2-Br-4,5-MDMA (12.5, 25, 50, and 100 μM) on: **(A)** collagen-induced platelet aggregation (platelet aggregation under saline without any drug was considered 100% aggregation), and **(B)** ATP released during collagen-induced platelet aggregation (ATP detected under saline without any drug was considered 100% release). Each bar represents a mean ± SEM (*n* = 3). Two-way ANOVA was used to identify the treatment (drug used) and/or the concentration (concentration used) as significant factors in the effect examined [for platelet aggregation, *F*_drug(1,__20)_ = 146 and *F*_concentration(4,__20)_ = 76.56; for ATP release, *F*_drug(1,__20)_ = 127.8 and *F*_concentration(4,__20)_ = 62.73]. Significance symbols arising from *post hoc* tests indicate either intragroup differences after 12.5, 25, 50, and 100 μM of drug concentration referred to saline controls (^∗^*p* < 0.05, ^∗∗^*p* < 0.01, ^∗∗∗^*p* < 0.001, Dunnett’s multiple comparisons test) or intergroup differences between the same concentrations of MDMA or 2-Br-4,5-MDMA (^##^*p* < 0.01, ^###^*p* < 0.001, Bonferroni multiple comparisons test).

Figure 2A shows that at 25 μM or higher, 2-Br-4,5-MDMA significantly inhibited collagen-induced platelet aggregation as compared to saline, whereas full inhibition was observed at 100 μM (^∗∗^*p* < 0.01, ^∗∗∗^*p* < 0.001, intragroup Dunnett’s multiple comparisons test). In contrast, MDMA induced only marginal (but significant) inhibition of platelet aggregation at 50 and 100 μM, an effect which was significantly weaker than that produced by 2-Br-4,5-MDMA (^###^*p* < 0.01, intergroup Bonferroni multiple comparisons test). Additionally, as seen in Figure 2B, both drugs exerted inhibitory effects upon the amount of ATP released from platelets (strong effect for 2-Br-4,5-MDMA and mild effect for MDMA, as compared to saline), in close parallelism to the effects of similar concentrations of the drugs on platelet aggregation (^∗∗∗^*p* < 0.01, for intragroup Dunnett’s multiple comparisons test; ^##^*p* < 0.01 and ^###^*p* < 0.001, for intergroup Bonferroni multiple comparisons test).

### Behavioral Evaluation

#### Effects of MDMA and 2-Br-4,5-MDMA on Spontaneous Motor Activity

**FIGURE 3:**
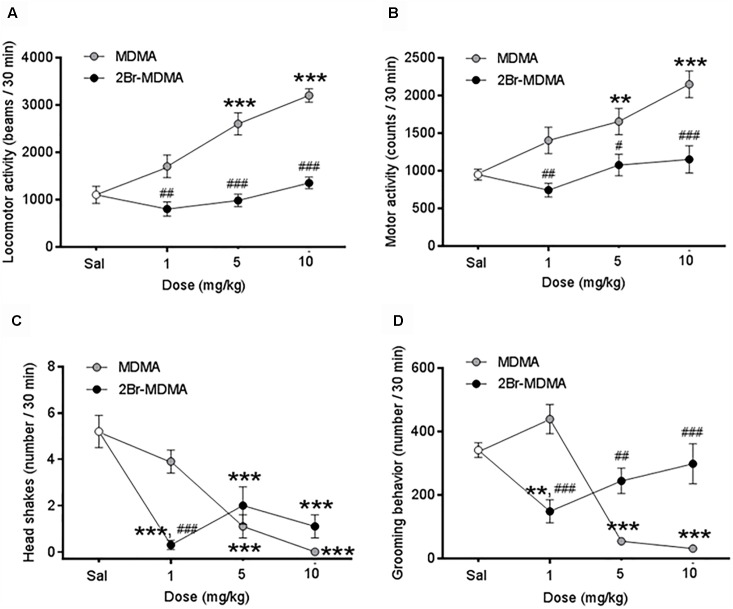
Dose-response curves for the effects of MDMA and 2-Br-4,5-MDMA on spontaneous motor behaviors of rats: **(A)** locomotor activity, **(B)** total motor activity, **(C)** head shakes, **(D)** grooming behavior. Saline (Sal) was used as control. Drugs (1, 5, and 10 mg/kg) or saline were injected i.p. in a total volume of 1 mL/kg body weight. Behaviors were scored during 30 min, starting 30 min after drug or saline administration. Each point represents a mean ± SEM (*n* = 8). Two-way ANOVA was used to identify the treatment (drug used) and/or the dose (dose used) as significant factors in the effect examined [for locomotor activity, *F*_drug(1,__56)_ = 75.71 and *F*_dose(2,__56)_ = 18.27; for total motor activity, *F*_drug(1,__56)_ = 29.97 and *F*_dose(2,__56)_ = 9.402; for head shakes, *F*_drug(1,__56)_ = 1.062 and *F*_dose(2,__56)_ = 26.70; for grooming behavior, *F*_drug(1,__56)_ = 2.689 and *F*_dose(2,__56)_ = 14.06]. Significance symbols coming from *post hoc* tests indicate either intragroup differences after 1, 5, or 10 mg/kg of drug referred to Sal controls (^∗∗^*p* < 0.01, ^∗∗∗^*p* < 0.001, Dunnett’s multiple comparisons test) or intergroup differences between the same doses of MDMA or 2-Br-4,5-MDMA (^#^*p* < 0.05, ^##^*p* < 0.01, ^###^*p* < 0.001, Bonferroni multiple comparisons test).

Figure 3A,B show that MDMA (5, 10 mg/kg) induced a significant increase in locomotor behavior and global motor activity compared to saline controls (^∗∗^*p* < 0.01, ^∗∗∗^*p* < 0.001, intragroup Dunnett’s multiple comparisons test), whereas 2-Br-4,5-MDMA did not induce changes in these motor parameters at any of the concentrations used. Differential sensitivity of locomotor and global motor behaviors to MDMA and 2-Br-4,5-MDMA administration was also detected upon comparison of the effects of both drugs at similar dosages (^#^*p* < 0.05, ^##^*p* < 0.01, ^###^*p* < 0.001, intergroup Bonferroni multiple comparisons test). Intragroup Dunnett’s *post hoc* comparisons showed a significant reduction in the number of head-shakes in MDMA-injected rats (5 and 10 mg/kg; ^∗∗∗^*p* < 0.0001) and also for those treated with any of the doses of the brominated analog (^∗∗∗^*p* < 0.001) (Figure 3C). Additionally, intragroup Dunnett’s comparisons indicated that MDMA (5 and 10 mg/kg) significantly reduced the grooming behavior (^∗∗∗^*p* < 0.0001), while at these doses 2-Br-4,5-MDMA did not induce any change in grooming activity (Figure 3D). In addition, neither MDMA nor 2-Br-4,5-MDMA altered rearing behavior, both being indistinguishable from controls (data not shown). Thus, as a whole, the foregoing experimental series indicate that except for head-shakes, 2-Br-4,5-MDMA lacks the distinctive psychomotor features of MDMA.

#### Effects of MDMA and 2-Br-4,5-MDMA in the Elevated Plus-Maze

**FIGURE 4:**
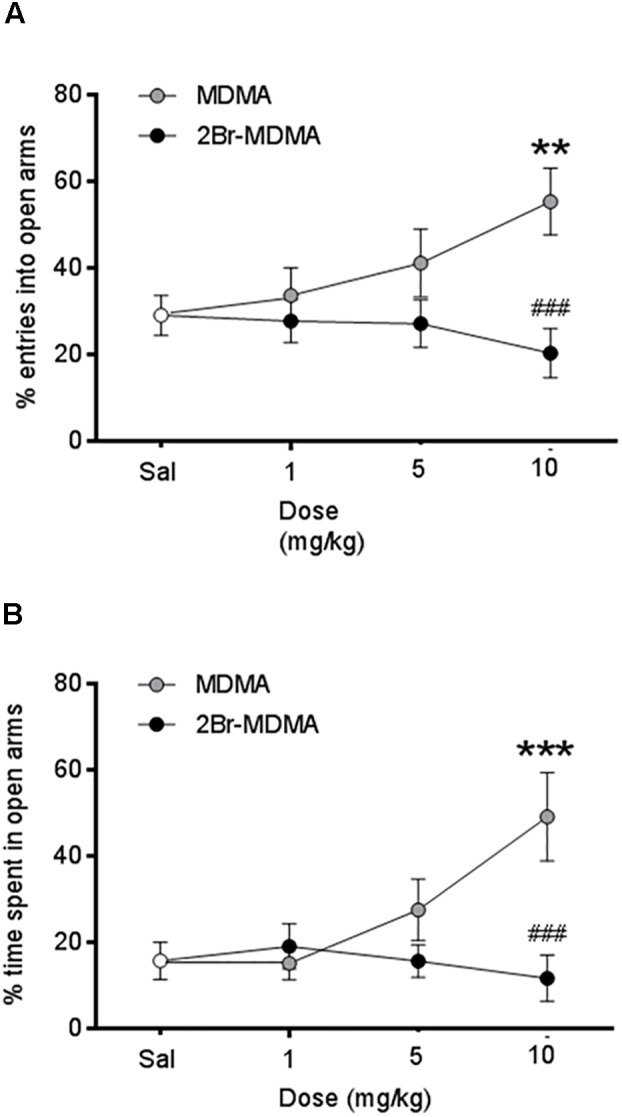
Dose-response curves for the effects of MDMA and 2-Br-4,5-MDMA on behavior of rats in the elevated plus maze: **(A)** % entries into open arms, **(B)** % time spent in open arms. Saline (Sal) was used as control. Drugs (1, 5, and 10 mg/kg) or saline were injected i.p. in a total volume of 1 mL/kg body weight. Behaviors were scored during 30 min, starting 30 min after drug or saline administration. Each point represents a mean ± SEM (*n* = 8). Two-way ANOVA was used to identify the treatment (drug used) and/or the dose (dose used) as significant factors in the effect examined [for % entries into open arms, *F*_drug(1,__56)_ = 10.33 and *F*_dose(2,__56)_ = 0.8359; for % time spent in open arms, *F*_drug(1,__56)_ = 7.432 and *F*_dose(2,__56)_ = 2.514]. Significance symbols arising from *post hoc* tests indicate either intragroup differences after 1, 5, or 10 mg/kg of drug referred to Sal controls (^∗∗^*p* < 0.01, ^∗∗∗^*p* < 0.001, Dunnett’s multiple comparisons test) or intergroup differences between the same doses of MDMA or 2-Br-4,5-MDMA (^###^*p* < 0.001, Bonferroni multiple comparisons test).

As seen on Figure 4, MDMA (10 mg/kg) induced a significant increase in the percentage of entries into the open arms (^∗∗^*p* < 0.01, intragroup Dunnett’s multiple comparisons test) and an increased percentage of time spent in the open arms as well (^∗∗∗^*p* < 0.001, intragroup Dunnett’s multiple comparisons test). In contrast, 2-Br-4,5-MDMA was unable to increase plus-maze open arms exploration at any of the doses used. On the other hand, the total number of entries into closed and open arms were not statistically different between 2Br-4,5-MDMA and MDMA, both being indistinguishable from controls (data not shown).

#### Effects of MDMA and 2-Br-4,5-MDMA on Active Avoidance Conditioning

**FIGURE 5:**
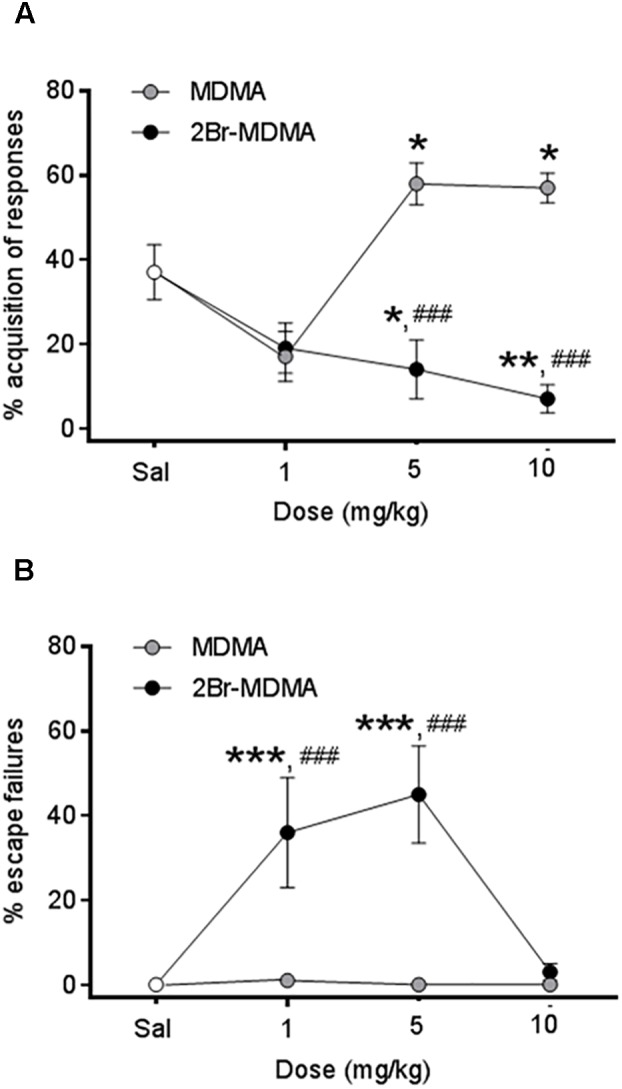
Dose-response curves for the effects of MDMA and 2-Br-4,5-MDMA on active avoidance conditioning behavior of rats: **(A)** % acquisition of responses, **(B)** % escape failures. Saline (Sal) was used as control. Drugs (1, 5, and 10 mg/kg) or saline were injected i.p. in a total volume of 1 mL/kg body weight. Behaviors were scored during 30 min, starting 30 min after drug or saline administration. Each point represents a mean ± SEM (*n* = 8). Two-way ANOVA was used to identify the treatment (drug used) and/or the dose (dose used) as significant factors in the effect examined [for conditioned avoidance responses, *F*_drug(1,__56)_ = 4.588 and *F*_dose(2,__56)_ = 11.96; for escape failures, *F*_drug(1,__56)_ = 22.55 and *F*_dose(2,__56)_ = 6.707]. Significance symbols arising from *post hoc* tests indicate either intragroup differences after 1, 5, or 10 mg/kg of drug referred to Sal controls (^∗^*p* < 0.05, ^∗∗^*p* < 0.01, ^∗∗∗^*p* < 0.001, Dunnett’s multiple comparisons test) or intergroup differences between the same doses of MDMA or 2-Br-4,5-MDMA (^###^*p* < 0.001, Bonferroni multiple comparisons test).

As seen in Figure 5A, intragroup Dunnett’s *post hoc* test reveals that whereas CAR was significantly improved after the administration of both higher doses of MDMA (^∗^*p* < 0.05), but it was significantly impaired by the same doses of 2-Br-4,5-MDMA (^∗^*p* < 0.05, ^∗∗^*p* < 0.01). On the other hand, Figure 5B shows that escape failures were not affected by MDMA, but they were increased in rats injected with 1 and 5 mg/kg of 2-Br-4,5-MDMA (^∗∗∗^*p* < 0.001, Dunnett’s multiple comparisons test). These results indicate that bromination of MDMA impairs the ability of the drug to improve acquisition in rats subjected to an active avoidance conditioning paradigm, together with producing an increase in escape failures.

#### Effects of MDMA and 2-Br-4,5-MDMA on Social Interaction

**FIGURE 6:**
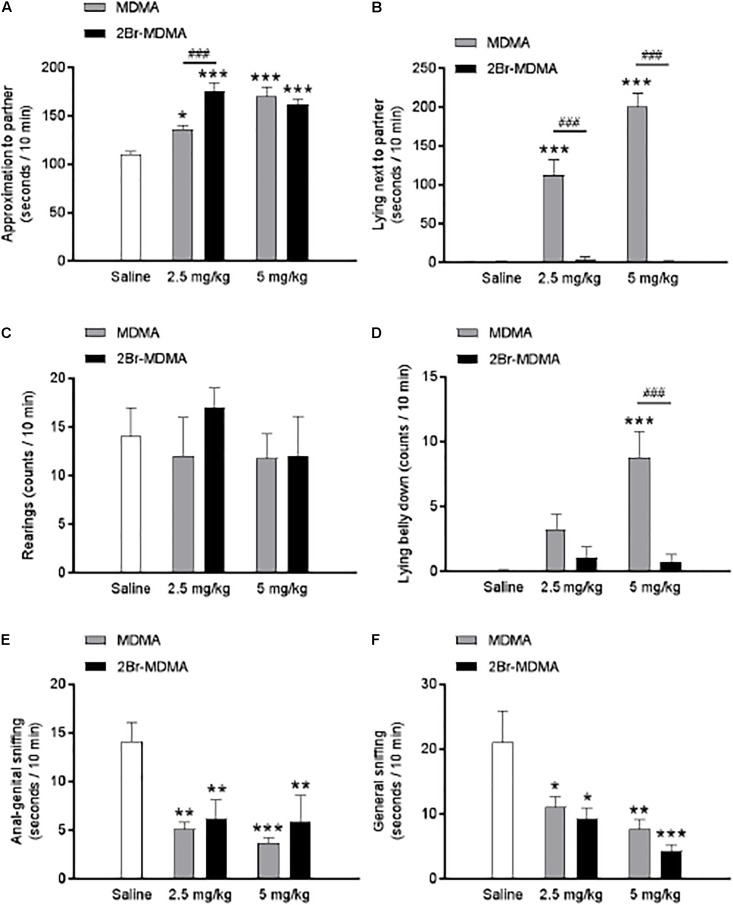
Dose-response relationships for the effects of MDMA and 2-Br-4,5-MDMA on social behavior of rats: **(A)** approaching the partner, **(B)** lying next to the partner, **(C)** standing on hindlimbs (rearing), **(D)** lying belly down, **(E)** anal-genital sniffing, **(F)** general sniffing. Saline was used as control. Drugs (1, 5, and 10 mg/kg) or saline were injected i.p. in a total volume of 1 mL/kg body weight. Behaviors were scored during 30 min, starting 30 min after drug or saline administration. Each bar represents a mean ± SEM (*n* = 8). Two-way ANOVA was used to identify the treatment (drug used) and/or the dose (dose used) as significant factors in the effect examined [for approaching the partner, *F*_drug(1,__42)_ = 4.004 and *F*_dose(2,__42)_ = 44.3; for lying next to the partner, *F*_drug(1,__42)_ = 127.7 and *F*_dose(2,__42)_ = 40.33; for standing on hindlimbs, *F*_drug(1,__42)_ = 0.4428 and *F*_dose(2,__42)_ = 0.3741; for lying belly down, *F*_drug(1,__42)_ = 16.04 and *F*_dose(2,__42)_ = 9.87; for anal-genital sniffing, *F*_drug(1,__42)_ = 0.5545 and *F*_dose(2,__42)_ = 16.15; for general sniffing, *F*_drug(1,__42)_ = 0.4586 and *F*_dose(2,__42)_ = 12.69]. Significance symbols arising from *post hoc* tests indicate either intragroup differences after 1, 5, or 10 mg/kg of drug referred to Sal controls (^∗^*p* < 0.05, ^∗∗^*p* < 0.01, ^∗∗∗^*p* < 0.001, Dunnett’s multiple comparisons test) or intergroup differences between the same doses of MDMA or 2-Br-4,5-MDMA (^###^*p* < 0.001, Bonferroni multiple comparisons test).

Dunnett’s *post hoc* test revealed that both MDMA and 2-Br-4,5-MDMA equally promoted closeness to the partner (^∗^*p* < 0.05, ^∗∗∗^*p* < 0.001) (Figure 6A), reduced anal-genital (^∗∗^*p* < 0.01, ^∗∗∗^*p* < 0.001) (Figure 6E) and general sniffing of the partner ^∗^*p* < 0.05, ^∗∗^*p* < 0.01, ^∗∗∗^*p* < 0.001) (Figure 6F), while both drugs produced no effect in the number of rearing episodes (Figure 6C). Nevertheless, MDMA administration significantly increased the time spent by rats lying next to each other and the lying belly down counts (^∗∗∗^*p* < 0.001) (Figure 6B,D), effects that were almost absent in rats receiving the brominated MDMA analog. Thus, the foregoing experimental series indicate that 2-Br-4,5-MDMA is almost unable to promote either the lying next to the partner response nor the lying belly down behavior, and did not significantly alter the other indexes of social interaction evaluated.

## Discussion

The central aim of the present work was to determine the affinity and activity at SERT of the brominated ecstasy analog 2-Br-4,5-MDMA, as well as its effects on the distinctive acute behavioral hallmarks of MDMA in classical spontaneous psychomotor models and social interaction in rats. The results obtained point to three major facts: first, aromatic bromination at C(2) preserves SERT affinity, increasing it about 10-fold compared to MDMA; second, 2-Br-4,5-MDMA fully inhibits platelet aggregation and ATP release during this process; third, 2-Br-4,5-MDMA lacks most of the typical MDMA-like effects in classical psychomotor models in rats. Indeed, none of the most relevant psychomotor responses for MDMA (increased locomotion, enhanced acquisition in active avoidance experiments, exploration in the plus-maze) are present (Quinteros-Muñoz et al., 2010). Moreover, and most interestingly, 2-Br-4,5-MDMA did not promote specific social behaviors (lying next to the partner, lying belly down behavior) that might be considered as the pharmacological signature of MDMA in the social interaction paradigm (Sáez-Briones and Díaz-Véliz, 2012). In contrast, for other behavioral responses that have been described as non-MDMA distinctive hallmarks (e.g., rearing, grooming, head shakes; Quinteros-Muñoz et al., 2010) no differences were observed between 2Br-4,5-MDMA and MDMA.

Seminal work carried out decades ago identified the vesicular and membrane-bound SERTs as major targets of non-exocytotic MDMA-mediated 5-HT release *in vitro*, as neurotransmitter efflux required Na^+^ on both sides of the membrane and was blocked by imipramine. Moreover, because these effects were reported to be different compared to those expected for structurally related psychedelics (Nichols and Glennon, 1984), it was proposed that the unique behavioral effects of MDMA might be mediated through its actions at SERT (Rudnick and Wall, 1992). Indeed, more recent evidence further elaborated that the acute psychotropic effects elicited by MDMA could be explained by means of a presynaptic mechanism that involves a reversal of the direction of transport of 5-HT through SERT elicited by the drug acting as a substrate (Sitte and Freissmuth, 2010; Dunlap et al., 2018).

The binding data for 2-Br-4,5-MDMA reported in the present work are in agreement with early *in silico* findings indicating that bromination at C(2) might not alter the binding capacity of MDMA (Núñez et al., 2009). Most interestingly, platelet aggregation inhibition data show that 2-Br-4,5-MDMA (but not MDMA) induced a fast and complete inhibition of platelet aggregation. Further, a mechanistic evaluation of the inhibition effects induced is consistent with the notion that 2-Br-4,5-MDMA might inhibit platelet aggregation by blocking the platelet serotonin resorption processes only, as also seems to be the case for the partial inhibition induced by MDMA (data not shown). A similar effect has already been reported for the SERT blocker citalopram at the same concentration using a similar experimental approach (Tseng et al., 2010). Nevertheless, considering the large differences among affinities at SERT, one may suggest that 2-Br-4,5-MDMA is a more potent blocker of serotonin influx than citalopram. The results obtained suggest that aromatic bromination at C(2) might modify the pharmacological profile of MDMA, favoring the establishment of blocking-like interactions at SERT that may account for the inhibition of platelet aggregation observed. In addition, although alterations in ATP release in the platelet model should be considered as an indirect measurement of possible differential functional activities at SERT (Tseng et al., 2010), these functional results for 2-Br-4,5-MDMA reinforce the idea of an apparent pharmacological similarity to citalopram. Indeed, aside from the concentration range considered, the inhibition of ATP release elicited by 2-Br-4,5-MDMA and citalopram reached almost the same maximum (Tseng et al., 2010). In contrast, the SERT substrate MDMA (which inhibits platelet aggregation by around 15% only) shows a modest inhibitory activity on ATP release in the same concentration range. Despite the latter, as ATP release inhibition should be considered as an indirect evidence of alterations in SERT functionality, experiments evaluating 5-HT release are required to characterize these effects further.

Until recently, it was proposed that SERT should possess different binding sites for substrates and inhibitors (Gabrielsen et al., 2012). Nevertheless, the X-ray structure of the human SERT bound to citalopram and paroxetine published recently differs in some extent from this model (Coleman et al., 2016). Indeed, the structure shows two binding sites: S1 (thought to be the common binding site for blockers) and S2 (allosteric or vestibular binding site), but crystallographic data indicate that citalopram may bind to both binding sites as part of a complex, stereospecific interaction process that accounts for its antidepressive effects that may be mechanistically different from other selective serotonin reuptake inhibitors such as paroxetine (Topiol et al., 2017). Interestingly, a similar mode of binding was proposed earlier for 5-HT and MDMA as well (Field et al., 2010). In this scenario, MDMA is proposed to share a binding pocket with 5-HT and to induce 5-HT efflux by a translocation process that is not fully understood (Sitte and Freissmuth, 2015). On the other hand, because citalopram binds at both S1 and S2, an interesting question to address might be if this mode of binding is shared by 2-Br-4,5-MDMA. If this were the case, a modified “binding site preference” might be associated with the alterations in the behavioral profiles compared to MDMA, possibly as a result of conformational changes associated with the transport process along the transporter passage. In this regard, an interesting possibility associated with the preference for S2 might be the occurrence of halogen bonding (Zhou et al., 2007) in addition to or as an alternative to steric and hydrophobic interactions. Further research is needed to consider these possibilities.

In order to address the complexity of the relationship between a presynaptic mechanism and its behavioral implications, most efforts have been focused on the elucidation of the molecular mechanism associated with transport reversal, mainly at human SERT. This critical event has been proposed to be responsible for the psychotropic effects elicited not only by MDMA at SERT but also by structurally related stimulants such as amphetamine and methamphetamine at the dopamine transporter (Sitte and Freissmuth, 2010, 2015). Moreover, a substrate-mediated neurotransmitter efflux is considered to be a distinctive pharmacological property of amphetamines, which differ in this regard from other non-amphetamine substrates (Hilber et al., 2005). The efflux is believed to be part of a more complex mechanism that includes a supplemental carrier trafficking induction associated with the alteration of presynaptic second messenger pathways. Therefore, critical interactions at SERT could be expected to be correlated with the differences among psychotropic amphetamines evidenced at the behavioral level. The results obtained in the present work agree with this assumption, as shown by the differences between MDMA and 2-Br-4,5-MDMA in key ecstasy-like behavioral paradigms. Regarding the latter, it should be noted that the behavioral characterization of ecstasy has been attempted in different animal models, producing heterogeneous results that depend on several factors, such as dosage regimen, animal species or administration routes (Sáez-Briones and Hernández, 2013; Dunlap et al., 2018). Nevertheless, and for the purpose of the present work, a behavioral profile of MDMA in rats after acute administration based on complete dose-effect curves under the same experimental conditions has been used to evaluate the influence of aromatic bromination (Quinteros-Muñoz et al., 2010).

Dose-dependent hyperlocomotion is one key classical behavior in the rat allowing MDMA and possible MDMA-like molecules to be distinguished from their stimulant and psychedelic congeners (Quinteros-Muñoz et al., 2010). Interestingly, in the present study bromine substitution abolished the ability of acutely administered MDMA to increase locomotion (already described by McCreary et al., 1999; Baumann et al., 2008), to enhance acquisition in active avoidance experiments (already described by Galizio et al., 2009) and exploration in the plus-maze (already described by Lin et al., 1999; Navarro and Maldonado, 2002), and to promote some specific social behaviors (already described by Morley and McGregor, 2000), suggesting that 2-Br-4,5-MDMA might be classified as non-MDMA-like. The latter might be supported indirectly, at least in part, by the pharmacological effects reported for synthetic cathinones (e.g., MDPV) that, acting as blockers of monoamine transporters, still exhibit psychomotor activity (Marusich et al., 2014).

In particular, it has already been proposed that the unique locomotion activity pattern induced by MDMA in rodents seems to be strongly dependent on the differential activation of central D_1_, D_2_, and D_3_ receptors (Risbrough et al., 2006). Therefore, one could speculate that the differences observed between MDMA and 2-Br-4,5-MDMA might be related to a differential activation rate of central dopaminergic receptors, as well as with the SERT and/or other monoamine transporters. The latter might be expressed as different availability ratios for dopamine and serotonin, as already reported for MDMA (Baumann et al., 2008). Interestingly, this effect seems to be dependent on the ability of MDMA to activate postsynaptic 5-HT_1B/1D_ and presynaptic 5-HT_2B_ receptors (Bankson and Cunningham, 2001; Doly et al., 2008) when administered acutely. As bromine substitution can be expected to modulate ligand affinity (Hardegger et al., 2010), binding experiments at 5-HT_1_ and 5-HT_2_ receptors with 2-Br-4,5-MDMA are required to correlate the absence of hyperlocomotion with a modulation of receptor affinity/activity induced by the presence of bromine. The latter may arise as a result of functional selectivity favoring alternative transduction pathways (Moya et al., 2007), a pharmacological property exhibited by structurally related halogenated hallucinogens that do not enhance locomotion (Quinteros-Muñoz et al., 2010).

Active avoidance conditioning responses represent another key model for the evaluation of MDMA-like molecules, because the behavioral profile of MDMA in this protocol may be differentiated from those of structurally related stimulants and psychedelics. Notably, as bromine substitution exhibits a new behavioral profile, not altering (at low doses) and disrupting (at 10 mg/kg) acquisition (together with an increase of the escape failures), one might classify these effects as mixed and/or psychedelic-like responses (Quinteros-Muñoz et al., 2010). As 2-Br-4,5-MDMA does not enhance the head shake response, one might speculate about the possibility that it might exhibit some hallucinogenic-like properties, as is the case of MMDA-2 that, by analogy with our naming of 2-Br-4,5-MDMA could be designated as 2-MeO-4,5-MDA (Shulgin and Shulgin, 1991; Quinteros-Muñoz et al., 2010). Interestingly, 2C-B (4-bromo-2,5-dimethoxyphenylethylamine), an entactogenic psychedelic (González et al., 2015) that does not enhance the head-shake response in rats and possesses low affinity for the 5-HT_2A_ receptor (Nelson et al., 1999) has been shown to be a low-affinity, non-competitive selective SERT blocker (Montgomery et al., 2007). The binding and functional data reported in the present work support this notion.

MDMA doses ranging from 2.5 to 5 mg/kg elevate “adjacent lying” in rats, a specific passive physical contact parameter measured in the social interaction model (Morley and McGregor, 2000; Ando et al., 2006). Pro-social behavior is mediated by the activation of serotonergic 5-HT_1A_ receptors elicited indirectly by acute doses of MDMA after massive 5-HT release in the hypothalamus (Morley et al., 2005) that elicits further the release of the neuropeptide oxytocin that, in turn, should be the direct effector of the pro-social behaviors induced by MDMA (Thompson et al., 2007). Interestingly, 2-Br-4,5-MDMA sustained almost all the pro-social behaviors typically enhanced by MDMA, with the only exception of the “adjacent lying” response. In this regard, one may speculate about a possible link between the amount of 5-HT released and the occurrence of specific pro-social behaviors. As 5-HT release should be modulated when affinity and/or activity at SERT changes, one might assume that bromine substitution diminished non-exocytotic 5-HT efflux, avoiding social interaction to be completed.

It should be noted that the entactogenic effects of MDMA in humans comprise a behavioral syndrome that may not be completely emulated in animal models. Nevertheless, social behavior has been used as a valuable tool in the preclinical testing of new drugs in laboratory animals. Indeed, an increase in social interaction has been shown to provide an index of potential anxiolytic activity (Cutier, 1993). This, in turn, establishes a link between social behavior and anxiety, highlighting the usefulness of this behavioral hallmark to estimate entactogenic-like effects (Sáez-Briones and Díaz-Véliz, 2012). Consequently, any disruption of basal anxiety may be expected to affect social behavior. As 2-Br-4,5-MDMA has been shown to be neither anxiolytic nor anxiogenic (Figure 4), one might propose that the selective abolishment of the “adjacent lying” response may not be related to an anxiety-related effect.

The question arises as to how bromination could disrupt the ability of MDMA to produce some specific behavioral effects while preserving (and even increasing) the capacity of the molecule to bind at SERT, as revealed by the nearly 10-fold lower K_i_ value of 2-Br-4,5-MDMA compared to MDMA. Two possible explanations may account for this apparent discrepancy: (i) firstly, bromination may have affected the ability of MDMA to cross the blood-brain barrier, thereby preventing 2-Br-4,5-MDMA from entering the brain, and (ii) secondly, bromination preserves the interaction of MDMA at SERT but may have altered the ability of MDMA to act as a substrate of the transporter (i.e., the ability to induce non-exocytotic 5-HT release). The first alternative seems to be unlikely considering that the bromine atom should increase the compound’s lipophilicity without greatly affecting its molecular bulk and weight and thus favor brain penetration. Instead, the second alternative is more plausible. The behavioral effects of a SERT substrate may be produced by a non-exocytotic 5-HT release that could induce the activation of serotonergic receptors in the brain. Therefore, the *in vivo* effects of MDMA bromination should be explained by a change in the molecular mechanism associated with transport reversal at SERT, eventually transforming 2-Br-4,5-MDMA into a SERT blocker. Further experiments to fully characterize this qualitative change in the mode of binding at SERT are needed to confirm this possibility.

Finally, and most interestingly, the results obtained in the present work indicate that the similarities between 2-Br-4,5-MDMA and citalopram offer some hints about the therapeutic potential of this new MDMA analog: on the one hand, its ability to act as a citalopram-like blocker of SERT suggests that it may act as an antidepressant; on the other hand, its strong inhibition of platelet aggregation might be consistent with an anticoagulant drug profile. These open possibilities deserve to be addressed further.

## Conclusion

In conclusion, and in contrast to our original hypothesis regarding the preservation or reinforcement of entactogenic qualities of MDMA, the results obtained in the present work are consistent with the notion that aromatic bromination of MDMA to give 2-Br-4,5-MDMA modulates its pharmacological features in a manner that resembles the effects of the SERT blocker citalopram, highlighting the relevance of this position on the aromatic ring as a key site to determine the mode of binding at SERT.

## Data Availability

The datasets generated for this study are available on request to the corresponding author.

## Author Contributions

PS-B provided ideas or concepts for definition of intellectual context, particularly designed, and supervised the experiments. VC-C and BC performed the organic synthesis of compounds. PS-B and GD-V performed the behavioral experiments. LV performed binding studies and experiments at SERT. RB and AH analyzed the data. PS-B wrote the manuscript. All authors read and approved the final version of the manuscript.

## Conflict of Interest Statement

The authors declare that the research was conducted in the absence of any commercial or financial relationships that could be construed as a potential conflict of interest.
